# Characterization of Renal Injury and Inflammation in an Experimental Model of Intravascular Hemolysis

**DOI:** 10.3389/fimmu.2018.00179

**Published:** 2018-03-01

**Authors:** Nicolas S. Merle, Anne Grunenwald, Marie-Lucile Figueres, Sophie Chauvet, Marie Daugan, Samantha Knockaert, Tania Robe-Rybkine, Remi Noe, Olivia May, Marie Frimat, Nathan Brinkman, Thomas Gentinetta, Sylvia Miescher, Pascal Houillier, Veronique Legros, Florence Gonnet, Olivier P. Blanc-Brude, Marion Rabant, Regis Daniel, Jordan D. Dimitrov, Lubka T. Roumenina

**Affiliations:** ^1^INSERM, UMR_S 1138, Centre de Recherche des Cordeliers, Paris, France; ^2^Sorbonne Universités, UPMC Univ Paris 06, Paris, France; ^3^Université Paris Descartes, Sorbonne Paris Cité, Paris, France; ^4^Assistance Publique – Hôpitaux de Paris, Service de néphrologie, Hôpital Européen Georges Pompidou, Paris, France; ^5^INSERM, UMR 995, Lille, France; ^6^University of Lille, CHU Lille, Nephrology Department, Lille, France; ^7^CSL Behring, R&D, Kankakee, IL, United States; ^8^CSL Behring, Research Bern, Bern, Switzerland; ^9^Université Paris-Saclay, CNRS, CEA, Univ Evry, Laboratoire Analyse et Modélisation pour la Biologie et l’Environnement, Evry, France; ^10^Paris Center for Cardiovascular Research, INSERM UMR_S 970, Paris, France; ^11^Assistance Publique – Hôpitaux de Paris, Service de pathologie, Hôpital Necker enfants malades, Paris, France

**Keywords:** hemolysis, heme, kidney injury, endothelial activation, inflammation, hemopexin, phenylhydrazine, experimental model of intravascular hemolysis

## Abstract

Intravascular erythrocyte destruction, accompanied by the release of pro-oxidative and pro-inflammatory components hemoglobin and heme, is a common event in the pathogenesis of numerous diseases with heterogeneous etiology and clinical features. A frequent adverse effect related to massive hemolysis is the renal injury and inflammation. Nevertheless, it is still unclear whether heme––a danger-associated molecular pattern––and ligand for TLR4 or upstream hemolysis-derived products are responsible for these effects. Well-characterized animal models of hemolysis with kidney impairment are needed to investigate how hemolysis drives kidney injury and to test novel therapeutic strategies. Here, we characterized the pathological processes leading to acute kidney injury and inflammation during massive intravascular hemolysis, using a mouse model of phenylhydrazine (PHZ)-triggered erythrocyte destruction. We observed profound changes in mRNA levels for markers of tubular damage (Kim-1, NGAL) and regeneration (indirect marker of tubular injury, Ki-67), and tissue and vascular inflammation (IL-6, E-selectin, P-selectin, ICAM-1) in kidneys of PHZ-treated mice, associated with ultrastructural signs of tubular injury. Moreover, mass spectrometry revealed presence of markers of tubular damage in urine, including meprin-α, cytoskeletal keratins, α-1-antitrypsin, and α-1-microglobulin. Signs of renal injury and inflammation rapidly resolved and the renal function was preserved, despite major changes in metabolic parameters of PHZ-injected animals. Mechanistically, renal alterations were largely heme-independent, since injection of free heme could not reproduce them, and scavenging heme with hemopexin in PHZ-administered mice could not prevent them. Reduced overall health status of the mice suggested multiorgan involvement. We detected amylasemia and amylasuria, two markers of acute pancreatitis. We also provide detailed characterization of renal manifestations associated with acute intravascular hemolysis, which may be mediated by hemolysis-derived products upstream of heme release. This analysis provides a platform for further investigations of hemolytic diseases and associated renal injury and the evaluation of novel therapeutic strategies that target intravascular hemolysis.

## Introduction

Intravascular erythrocyte destruction, accompanied by the release of pro-oxidative and pro-inflammaotry components hemoglobin and heme, is a common event in the pathogenesis of numerous diseases with heterogeneous etiologic factors and clinical features, such as sickle-cell disease (SCD), microangiopathic hemolytic anemias, ABO mismatch transfusion reaction, paroxysmal nocturnal hemoglobinuria, autoimmune hemolytic anemia, malaria, cardiopulmonary bypass, mechanical heart valve-induced anemia and chemical-induced anemias, and many others (1). Excessive or chronic intravascular hemolysis may overwhelm the protective, scavenging system made of haptoglobin, and hemopexin (Hx) in plasma, and lead, in a fraction of the patients to common lesions, one being renal injury. Adverse clinical effects related to free hemoglobin and heme release can be caused by direct cytotoxicity, nitric oxide scavenging and vasoconstriction, inflammation, and oxidative reactions (including lipid peroxidation and mitochondrial dysfunction) (2–6). Heme exerts direct cytotoxic effects, drives pro-oxidative, pro-thrombotic and pro-inflammatory effects on endothelium, and induces immune cell activation (7). Free hemoglobin and free heme have multiple effects on plasma systems [complement (8), coagulation (9), antibodies (10)], on endothelium (11, 12) and tissues, and particularly in organs like kidneys (13).

Intravascular hemolysis is associated with acute kidney injury, most likely due to oxidative stress, cytotoxicity resulting in tubular necrosis, intratubular casts, and pro-inflammatory effects, such as production of IL-6 or MCP-1 (13–16). To understand to what extend the hemolysis induces kidney injury, what are the underlying mechanisms of these processes and to test novel therapeutic strategies, animal models of hemolysis with well-characterized kidney manifestations are needed. Several animal models have been set up to study hemolysis, but kidney function was not always fully described. One of the most frequently used methods to trigger intravascular hemolysis in experimental animals is the injection of a hemolytic drug phenylhydrazine (PHZ). PHZ induces massive intravascular hemolysis by lipid peroxidation of erythrocyte membranes (17), destabilizing the globin portion of hemoglobin, leading to its denaturation, precipitation, and the release of globin-free heme. PHZ is good model for studying acute phase or chronic intravascular hemolysis (18, 19).

The objective of this study is to characterize the pathological processes leading to kidney injury in acute drug-induced intravascular hemolysis. We aimed to characterize the inflammation and the renal phenotype of a mouse model of intravascular hemolysis triggered by PHZ and to find out to what extend it is dependent on the release of free heme.

## Materials and Methods

### Reagents

Solution of PHZ of 25 mg/mL (Sigma-Aldrich) was prepared in PBS immediately before use. The Fe^3+^ form of heme [hemin (ferriprotoporphyrin IX), designated as heme (Frontier Scientific Inc. or Sigma-Aldrich)] was dissolved to 20 mM in 50-mM NaOH and 145-mM NaCl, and further diluted in PBS just before use. Plasma-purified Hx was provided by CSL Behring.

### Mouse Treatment

All experiments were conducted in accordance with the recommendations for the care and use of laboratory animals following the ARRIVE regulations and with the approval APAFIS#3764-201601121739330v3 of the French Ministry of Agriculture. C57Bl/6 mice were from Charles River Laboratories (L’Arbresle, France). Female C57Bl/6 mice were injected i.p. with 200 µL of PBS (Gibco), or freshly prepared heme [40 µmol/kg, corresponding to 28-µg/g body weight (20)] repeated 24 h later (days 0 and 1). PHZ (900 µmol/kg, corresponding to 0.125-mg/g body weight) was injected i.p. in at day 0 and C57Bl/6 mice sacrificed by cervical decerebration at days 1, 2, or 4 at the age of 8 weeks. Only single-sex mice were used for consistency and female were selected since they are less aggressive. To test the efficacy of Hx, mice were pretreated with i.p. injection of 40 µmol/kg of human Hx 1 h before i.p. injection of 900 µmol/kg of PHZ. Concentrations and route of administration were chosen as described (19–21). Mice kidneys were recovered and snap-frozen in liquid nitrogen or fixed and included in paraffin.

### Evaluation of the Renal Function

The follow-up of renal function was performed by placing 6-week-old C57Bl/6 female mice (Charles Rivers) in individual metabolic cages for acclimation for 5 days, with daily weight measurement and fixed, daily amounts of food. After stabilization of body weight (day −3) metabolic parameters were measured (diuresis, amount of excrements, water and food uptake, and body weight). Urine was collected at fixed times for 3 days at baseline, and 2 days after injections. PHZ, heme, or PBS were injected at day 0. Blood was taken from the retro-orbital sinus before and at day 2 after treatment. Blood gas was measured at day 2 after treatment (Alere system, self-calibrated epoc^®^ BGEM Test Card). Organs were recovered at day 2. In alternative experiments in regular cages organs were recovered at day 4. Organs were snap-frozen in liquid nitrogen in Cryomatrix (Thermoscientific). Urinary protein and creatinin levels, as well as plasma urea concentrations were measured using Konelab equipment. For comparative purposes, similar experiments were performed in regular cages, with five animals per group.

### Immunohistochemistry on Mouse Tissues

Moreover, 3-μm-thick sections of fixed (PFA 4%) frozen kidneys were cut with Cryostat Leica AS-LMD. Heme oxygenase 1 (HO-1) expression was studied using rabbit anti-mouse HO-1 (Abcam, Ab13243) followed by a polymer anti-rabbit IgG-HRP (DAKO, K4003). Staining was revealed with DAB solution. Slides were scanned by Nanozoomer (Hamamatsu). Hematoxylin–Eosin, Perl’s Prussian blue, and PAS coloration were performed by routine procedures using sections of paraffin-embedded kidneys at days 1, 2, and 4. Coloration of slides was scanned by Slide Scanner Axio Scan (Zeiss).

### mRNA Level Analyses

Snap-frozen kidney sections were recovered in RLT buffer (Qiagen) + 1% β-mercaptoethanol (Gibco) and used for mRNA extraction with Qiagen RNeasy miniKit. The quality and quantity of mRNA were evaluated with bioanalyser Agilent 2100 using Agilent TNA 6000 NanoKit and if the RNA integrity number was >7 the mRNA was retrotranscribed to cDNA. Gene markers of early tubular and endothelium activation/injury relevant for hemolysis and SCD were analyzed by low-density array (LDA, ThermoFisher) including NGAL, Kim-1, HO-1, Il-6, Ki67, ICAM-1, E-selectin and P-selectin, Caspase-3, and CD31 (16, 22–25), and validated by RTqPCR for NGAL, Kim-1, HO-1, and Ki67 (ThermoFisher). Il-1β and TNF-α have been tested by RTqPCR (ThermoFisher).

### Urinary Electrophoresis

Urine of the mice recovered from the metabolic cages was centrifuged and diluted 1/2 with a sample buffer containing glycerol and bromophenol blue (but not SDS) and 20 µL are deposited on 10 wells 12% gels and migrated for 40 min. Staining the gel-resolved proteins was performed with Coomassie blue.

### Mass Spectrometry Analyses

After discoloration, bands of interest were excised from the Coomassie blue-stained SDS page gel at the same level for the PBS, heme and PHZ-treated animals. Samples were then reduced with the addition of DTT (4.2 mM, final concentration) for 45 min at 37°C, then alkylated with iodoacetamide (7.6 mM, final concentration) and subjected to in-gel tryptic digestion using porcine trypsin (Promega, France) at 12.5 ng/µL. The dried peptides extracts were dissolved in 12 µL of solvent A (2% acetonitrile, 0.1% formic acid) and analyzed by online nanoLC using an Ultimate 3000 System (Dionex) coupled to a LTQ-XL Orbitrap mass spectrometer (Thermo Fisher Scientific). Each peptide extract (5 µL) was loaded on a C18 precolumn (Acclaim PepMap C18, 5-mm length × 300-µm I.D., 5-µm particle size, 100-Å porosity, Dionex) at 20 µL/min in solvent A. After 5-min desalting, the precolumn was switched online with a C18 capillary column (Acclaim PepMap C18, 15-cm length × 75-µm ID × 3-µm particle size, 100-Å porosity, Dionex) equilibrated in solvent A. Peptides were eluted using a 0–70% gradient of solvent B (80% acetonitrile, 0.1% formic acid) during 50 min at a flow rate of 300 nL/min. The LTQ-XL Orbitrap was operated in data-dependent acquisition mode with the XCalibur software. Survey scan MS were acquired in the Orbitrap in the 400–1,600 *m*/*z* range with the resolution set to a value of 60,000. The five most intense ions per survey scan were selected for collision-induced dissociation (CID) fragmentation and the resulting fragments were analyzed in the linear trap (LTQ). Dynamic exclusion was employed within 45 s to prevent repetitive selection of the same peptide.

Peak lists extraction from XCalibur raw files were automatically performed using Proteome Discover software (version 1.4, Thermo Fisher scientific). Database searches were performed using the Mascot server v2.2.07 with the following parameters: database *Mouse*; enzymatic specificity: tryptic with two allowed missed cleavages; fixed modification of cysteine residues [carbamidomethylated (C)]; variable oxidation of methionine residues; 5-ppm tolerance on precursor masses and 0.6-Da tolerance on fragment ions; fragment types taken into account were those specified in the configuration “ESI-trap.”

### Western Blotting

The gels with urine proteins, resolved by electrophoresis as above were transferred to nitrocellulose membranes using iBlot equipment (Invitrogen) and stained with Rabbit polyclonal anti-pancreatic alpha amylase antibody (Abcam, ab199132) using SNAP technology (Merk Millipore), followed by immunodetection with Goat anti Rabbit IgG-HRP (Santa Cruz, 1/5,000). The signal was revealed by chemiluminescence.

### Quantification of Plasmatic Levels of Alpha Amylase

The levels of α-amylase were detected in mouse plasma, using Colorimetric Amylase Assay Kit (Abcam, ab102523) using the protocol provided by the manufacturer.

### Activation of Endothelial Cells by PHZ

Primary human umbilical vein endothelial cells (HUVEC) were cultured as described previously (8, 26) and treated with decreasing doses of PHZ: 2.5, 1.25, 0.625, 0.312, 0.156, 0.078, 0.039, 0.019 mg/mL, or TNF-α 10 ng/mL or medium only for 24 h. Cells in 24-well plates were detached with trypsine and washed with cold PBS. The cells were resuspended in 300 µL of PBS BSA 2%, dispatched in three tubes and labeled with anti-E-selectine antibody 1:50 (Immunotech, 1243) or mouse anti-VCAM1 FITC antibody 1:50 (AbD serotec, 0770) or mouse anti-ICAM-1 antibody 1:50 (Beckman, IM0544) or anti-P-selectin antibody (BIORAD, MCA796) for 30 min at 4°C. For the ICAM-1 and P-selectin staining, a goat anti-mouse PE antibody 1:100 (Beckman, IM0855)was used for 30 min at 4°C. After washing, the cells were resuspended in 100 µL of annexin-V-binding buffer (BD, 556454) and labeled with annexin-V-APC (1:100) (BD, 550474) for 15 min at room temperature and DAPI (1:100). After the incubation, 400 µL of annexin buffer was added before flow-cytometry analysis (FACS LSR II).

### Statistics

Results were analyzed using a statistical software package (GraphPad Prism 5) as indicated in figures legends. Briefly, * p < 0.05, ** *p* < 0.005, and *** *p* < 0.001. Multiple groups in the mRNA level analyses were compared with Kruskal–Wallis with Dunn’s test for multiple pairwise comparisons. When only two groups were compared, Mann–Whitney test was applied. The metabolic parameters were compared for the mice before and after indicated treatments using two-way ANOVA with Tukey’s test for multiple pairwise comparisons.

## Results

### Characterization of the Kidney Function in the PHZ-Induced Intravascular Hemolysis Model

**Figure 1:**
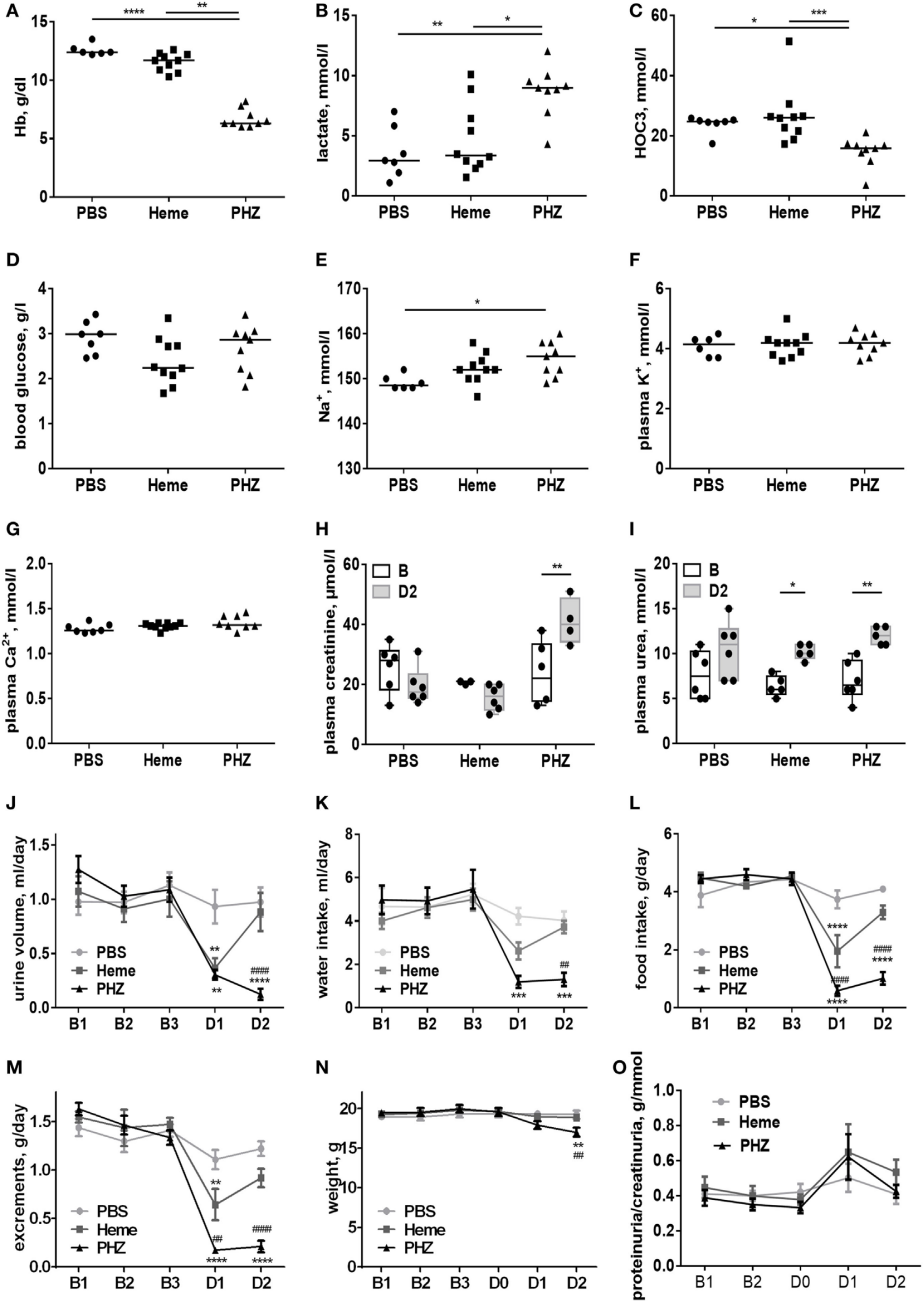
Evaluation of the renal function parameters in the phenylhydrazine (PHZ) and heme-injected mice in metabolic cages. **(A)** Blood total hemoglobin, **(B)** plasma lactate, **(C)** plasma HCO_3_^−^, **(D)** blood glucose, **(E)** plasma sodium, **(F)** plasma potassium, **(G)** plasma calcium, **(H)** plasma creatinine, and **(I)** plasma urea. * *p* < 0.05, ** *p* < 0.005, *** *p* < 0.001, and Kruskal–Wallis with Dunn’s test for multiple pairwise comparisons **(A–G)** or performed by two-way ANOVA with Tukey’s test for multiple pairwise comparisons **(H,I)**. Kinetic evolutions of **(J)** urine volume, **(K)** water intake, **(L)** food intake, **(M)** excrements, **(N)** weight, and **(O)** proteinuria/creatininuria ratio. B1, B2, and B3 correspond to baseline measures three consecutive days before injection, and D1 and D2 correspond to mice follow-up two consecutive days after i.p. injection (for each measure, *n* ≥ 6 per group). ** *p* < 0.005, *** *p* < 0.001, and **** *p* < 0.0001 compared with PBS-treated mice, ^##^ *p* < 0.005 and ^####^ *p* < 0.0001 compared with heme-treated mice, and two-way ANOVA with Tukey’s test for multiple pairwise comparisons.

To find out to what extend the acute intravascular hemolysis and heme induce renal injury, we evaluated the parameters of the kidney function, placing the mice in metabolic cages. Blood gas analyses revealed that heme- and PHZ-treated mice had decreased blood hemoglobin at day 2 compared with PBS controls of about 8% (not reaching significance) and significant 46%, respectively (Figure 1A). This erythrocytes lysis was associated with a significant increase in lactate as a surrogate marker of hypoxia and cell damage (Figure 1B) and a decrease in the HCO_3_^−^, a marker of metabolic acidosis (Figure 1C) in the PHZ group. The heme-treated group presented with slightly decreased blood glucose at day 2, without reaching significance (Figure 1D). Plasma Na^+^ was increased in the PHZ-treated groups (Figure 1E), while K^+^ (Figure 1F) and Ca^2+^ (Figure 1G) remained unaltered. Furthermore, we measured urea and creatinine to estimate the changes in glomerular filtration rate. Plasma creatinine concentration (Figure 1H) and the plasma urea (Figure 1I) were significantly increased in PHZ-treated mice compared with the baseline. Moreover, heme treatment also increased the plasma urea in a significant manner compared with baseline (Figure 1I). PHZ- and heme-injected mice also presented with an oliguria (24-h diuresis) at day 1 (Figure 1J) which persisted in day 2 for PHZ but normalized for heme-injected mice. These were accompanied by reduced water (Figure 1K) and food (Figure 1L) intake, resulting in a decrease of excrements (Figure 1M) and 13% weight loss at day 2 for the PHZ-injected mice (Figure 1N). Proteinuria normalized by the creatininuria was not significantly different in PHZ- and heme-treated mice compared with PBS group (Figure 1O). Alteration of kidney function parameter is most likely functional, due to the anemia and reduced food and water uptake and less likely due to renal tissue damages.

### Histological Analyses of Mouse Kidneys Do Not Reveal Major Structural Alterations

**Figure 2:**
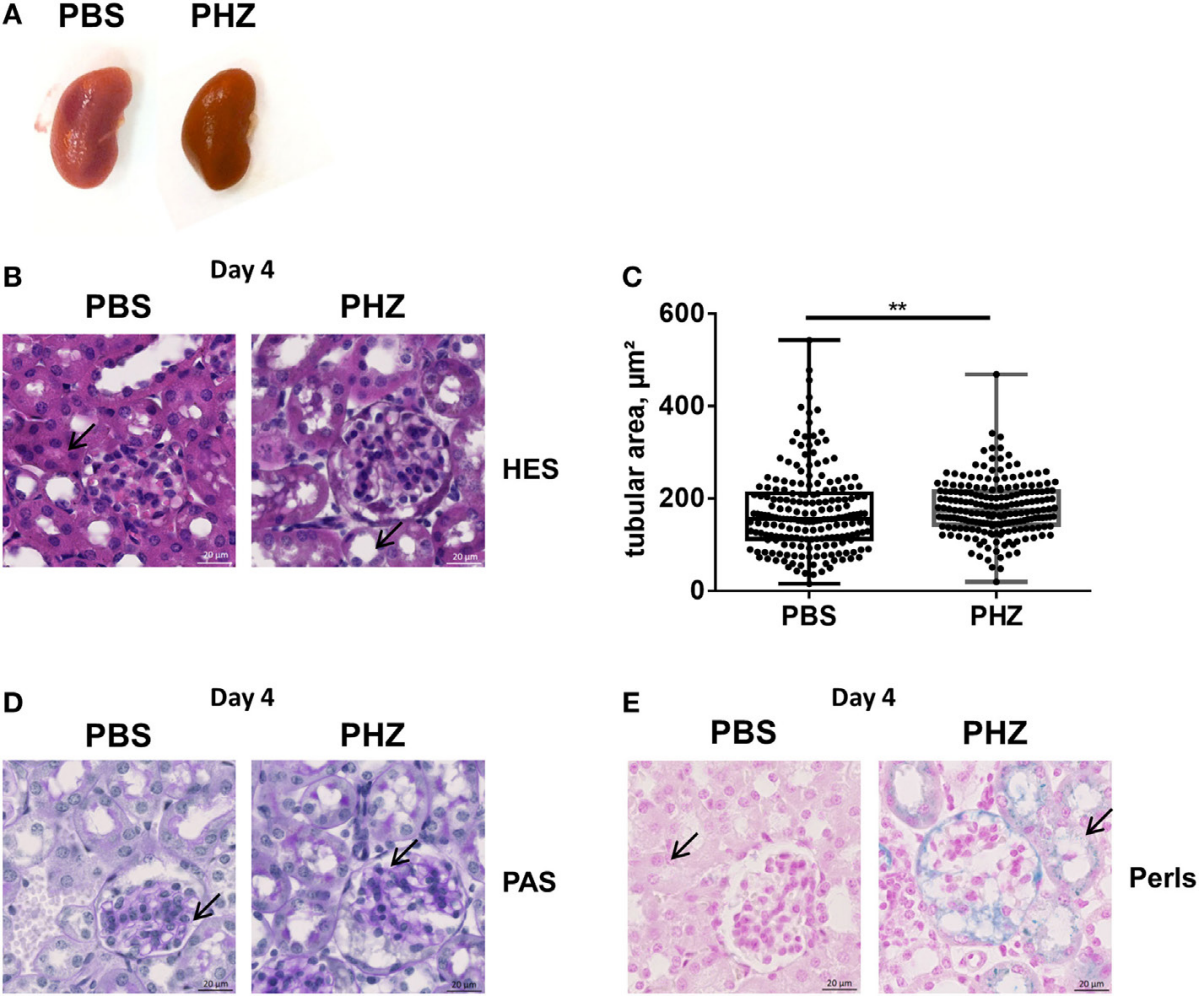
Renal histology of phenylhydrazine (PHZ)-treated mice. **(A)** Representative images of kidneys from PBS- and PHZ-treated mice. **(B)** Fixed and paraffin-embedded kidneys from mouse sacrificed at day 4 were cut at 3 µm and stained with hematoxylin–eosin (×15). **(C)** Quantitative analysis of proximal tubular size; each point represents the size of one tubule (*n* = 200). Mean ± SD, ** *p* < 0,005, and Mann–Whitney test. **(D,E)** Fixed and paraffin-embedded kidneys (×20) from mice sacrificed at day 4 were cut at 3 µm and stained with PAS **(D)**, Perl’s Prussian Blue **(E)**. Black arrows point to tubules lumen **(B)**, to glomerular mesangium **(D)**, or to iron deposition **(E)** in PBS- and PHZ-treated mice.

Kidneys (Figure 2A), blood as well as liver and spleen of PHZ-treated mice were dark in color, compared with PBS-injected controls, while heme-treated mice showed only mild dark coloration. To gain a deeper insight whether acute hemolysis induced tissue injury in the kidney, renal sections were analyzed by histology. By light microcopy, paraffin-embedded kidney sections of the mice treated with PBS or PHZ were similar regarding the analysis of glomeruli, that were normal with no modification of glomeruli size, endocapillary proliferation, glomeruli were not congested, and did not shown neither TMA lesions nor mesangial proliferation at day 2 (not shown) and day 4 (Figures 2B,C). No major modification of renal histology was detected, except minor but significant increase of tubular dilatation at day 4, a sign of acute tubular necrosis at day 4 (*p* = 0.009) (Figures 2B,C). No signs of immune cells infiltration were detected by histology at days 1, 2 (data not shown), or 4 (Figures 2B,D) and CD45 staining (data not shown). Perl’s Prussian blue staining for hemosiderin, a hallmark for hemolysis, was positive in the PHZ-injected mice (Figure 2E).

### Hemolysis-Induced Upregulation of Tissue Injury and Inflammation-Related Genes in the Kidney in a Heme-independent Manner

**Figure 3:**
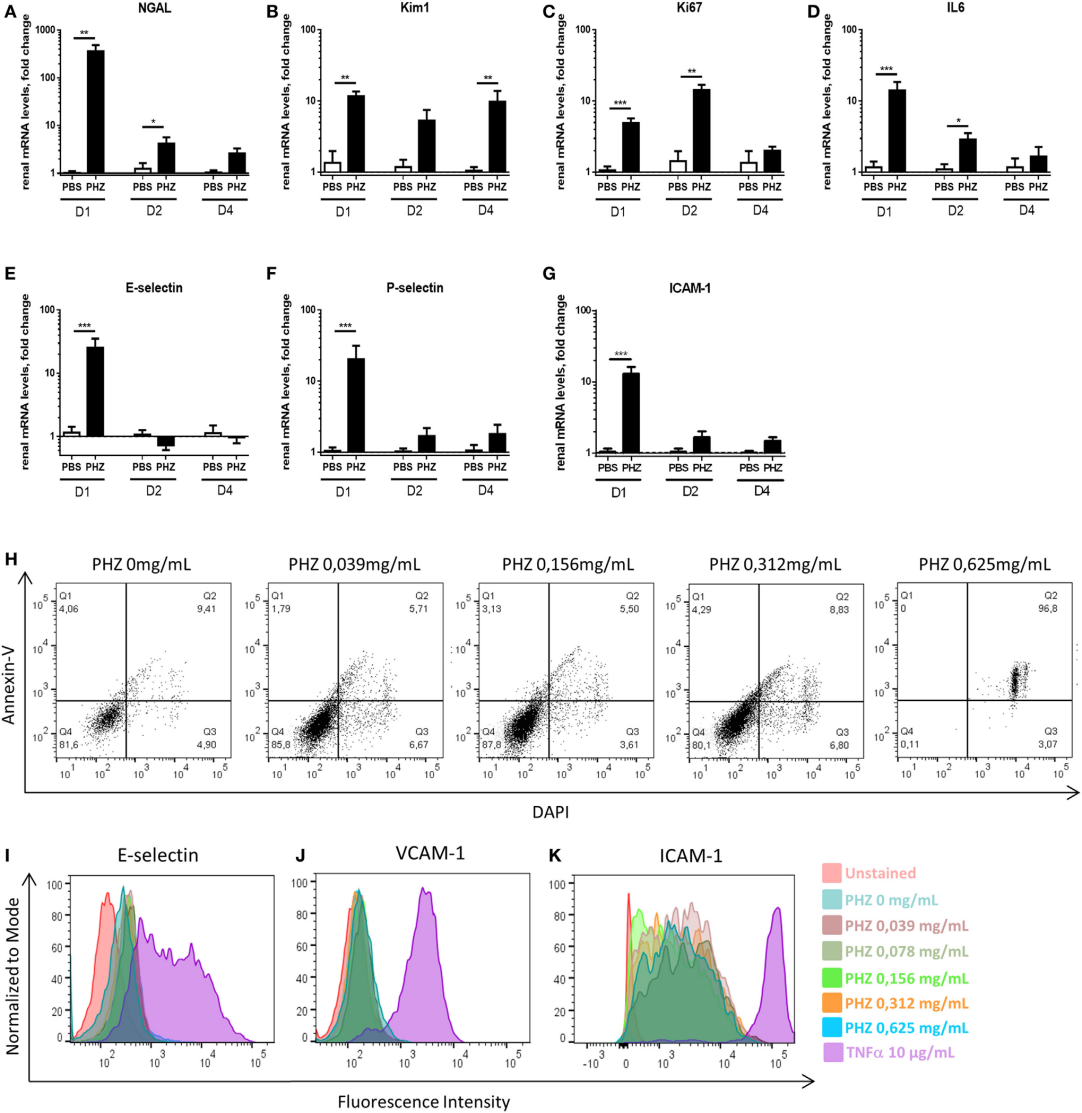
Hemolysis inducing renal injury. Kinetics of mRNA levels in renal tissue of **(A)** NGAL, **(B)** Kim-1, **(C)** Ki-67, **(D)** IL-6, **(E)** E-selectin, **(F)** P-selectin and **(G)** ICAM-1 at 1, 2, and 4 days after PBS or phenylhydrazine (PHZ) injection. **(H)** Human umbilical vein endothelial cells (HUVECs) were incubated for 24 h with increased concentration of PHZ in M199 medium, containing 20% fetal calf serum, and cell death was measured by double staining with annexin-V and DAPI by flow cytometry. **(I–K)** HUVECs were incubated for 24 h with increased concentration of PHZ or TNF-α as a positive control, in M199 medium, containing 20% fetal calf serum. E-selectin **(I)**, VCAM-1 **(J)**, and ICAM-1 **(K)** were measured by flow cytometry. Data are presented for 0.312-mg/mL PHZ, gating on live (annexin-V^−^, DAPI^−^ cells, about 80% of the total cell population). Mean ± SEM, * *p* < 0.05, ** *p* < 0.005, *** *p* < 0.001, and Mann–Whitney test.

Furthermore, we analyzed at gene level a number of more sensitive markers for inflammation and tissue injury in the acute hemolysis model. Sustained expression of markers of tubular injury NGAL and Kim-1, till days 2 and 4, respectively (Figures 3A,B), and sustained expression of markers of proliferation––Ki67 and inflammation––IL-6 till day 2 (Figures 3C,D) were detected. How ever, endothelial activation E-selectin, P-selectin, and ICAM-1 (Figures 3E–G) were detected only at day 1. No difference in immune cell-produced inflammation mediators IL-1b and TNF-α, apoptosis marker Caspase-3 as well as endothelial cell junction factor CD31 was detected (not shown).

To find out whether the endothelial activation was due to intravascular hemolysis or related to direct effects of PHZ, endothelial cells (HUVEC) were exposed to PHZ *in vitro* for 2 h (the duration of the *in vivo* experiment). Increasing doses up to 0.312 mg/mL did not promote apoptosis (over 80% of the cells being annexin-V^−^ DAPI^−^), while higher doses resulted in a massive cell death (Figure 3H). In addition, incubation of PHZ for 24 h did not induce cell-surface expression of adhesion markers such as E-selectin, VCAM-1, or ICAM-1, contrary to 10 µg/mL of TNF-α (Figures 3I–K). Neither PHZ nor TNF-α triggered surface expression of P-selectin (not shown).

**Figure 4:**
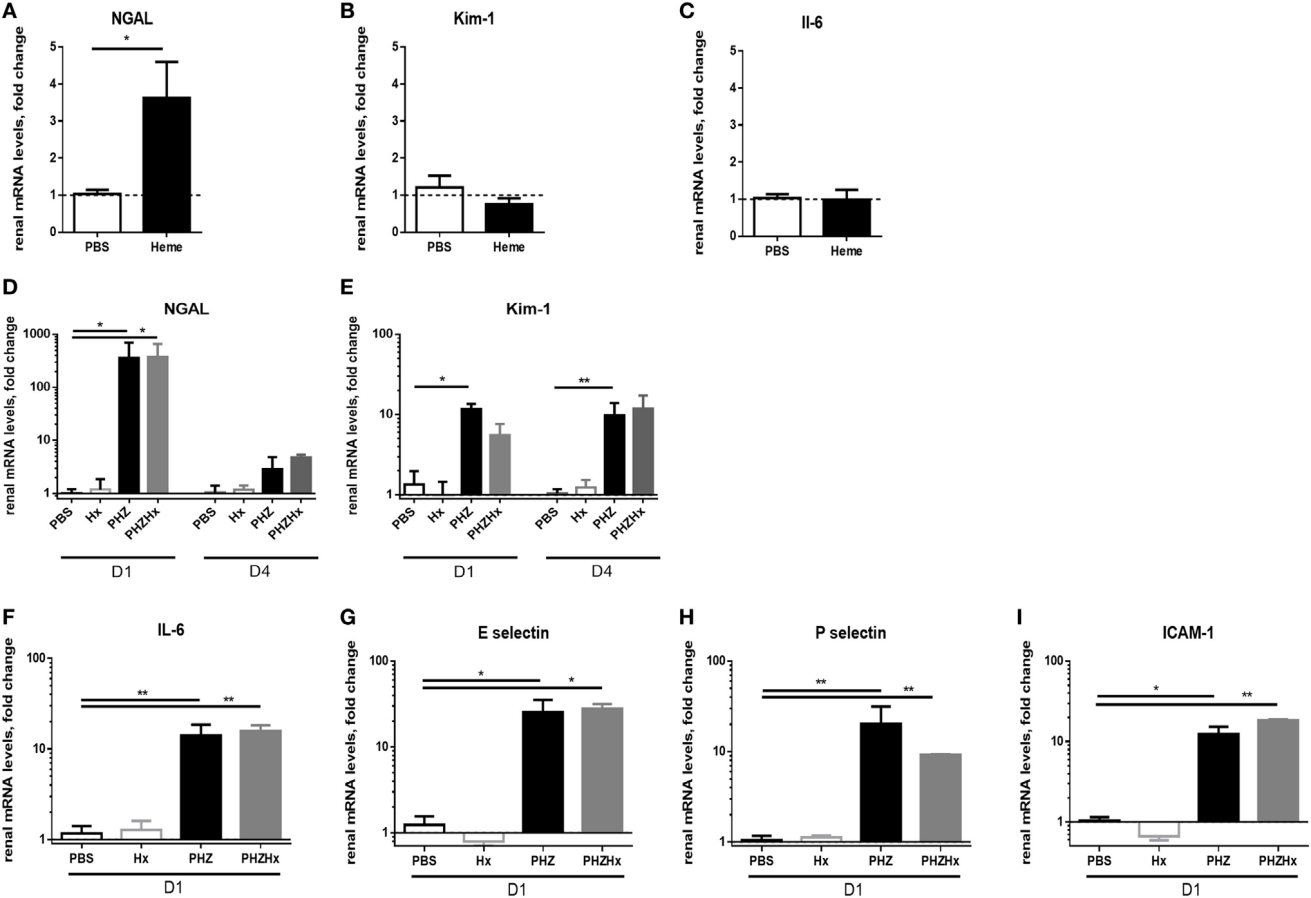
The renal injury which is largely heme-independent. mRNA levels of NGAL **(A)**, Kim-1 **(B)**, and IL-6 **(C)** in renal tissue after injection of heme at day 2. Influence of hemopexin (Hx) on mRNA level in the phenylhydrazine (PHZ) model, **(D)** NGAL, **(E)** Kim-1, **(F)** IL-6, **(G)** E-selectin, **(H)** P-selectin, and **(I)** ICAM-1 at day 1 or day 4, as mentioned under the panels. * *p* < 0.05, ** *p* < 0.005, Mann–Whitney test for **(A–C)**, and Kruskal–Wallis with Dunn’s test for multiple pairwise comparisons for **(D–I)**. Panels **(A–C)** compare genes expression after two injections of heme at day 0 and day 1. Panels **(D,E)** compare the genes expression at days 1 and 4 (D1 and D4) after injection of PHZ ± Hx. The remaining panels show results for D1 after injection of PHZ ± Hx.

To find out whether these changes are heme-dependent or occur due to upstream products (as hemoglobin), we investigated expression of NGAL, Kim-1, and IL-6 in heme-treated mice at day 2. We detected a significant increase of NGAL expression (Figure 4A), about 50% weaker compared with NGAL expression in PHZ-treated mice at day 2. However, no modification of mRNA levels of Kim-1 and IL-6 (Figures 4B,C) were detected. Furthermore, the i.p. injection of the heme scavenger human plasma-derived Hx did not prevent the expression of these genes after PHZ treatment (Figures 4D–I), showing a largely heme-independent process.

We analyzed the expression of the same gene panel in liver and spleen and observed similar alterations of a set of genes in the PHZ-treated mice, indicating that this model is associated with multiorgan lesions (data not shown).

### Renal Tubules Upregulating HO-1 after Exposure to PHZ

**Figure 5:**
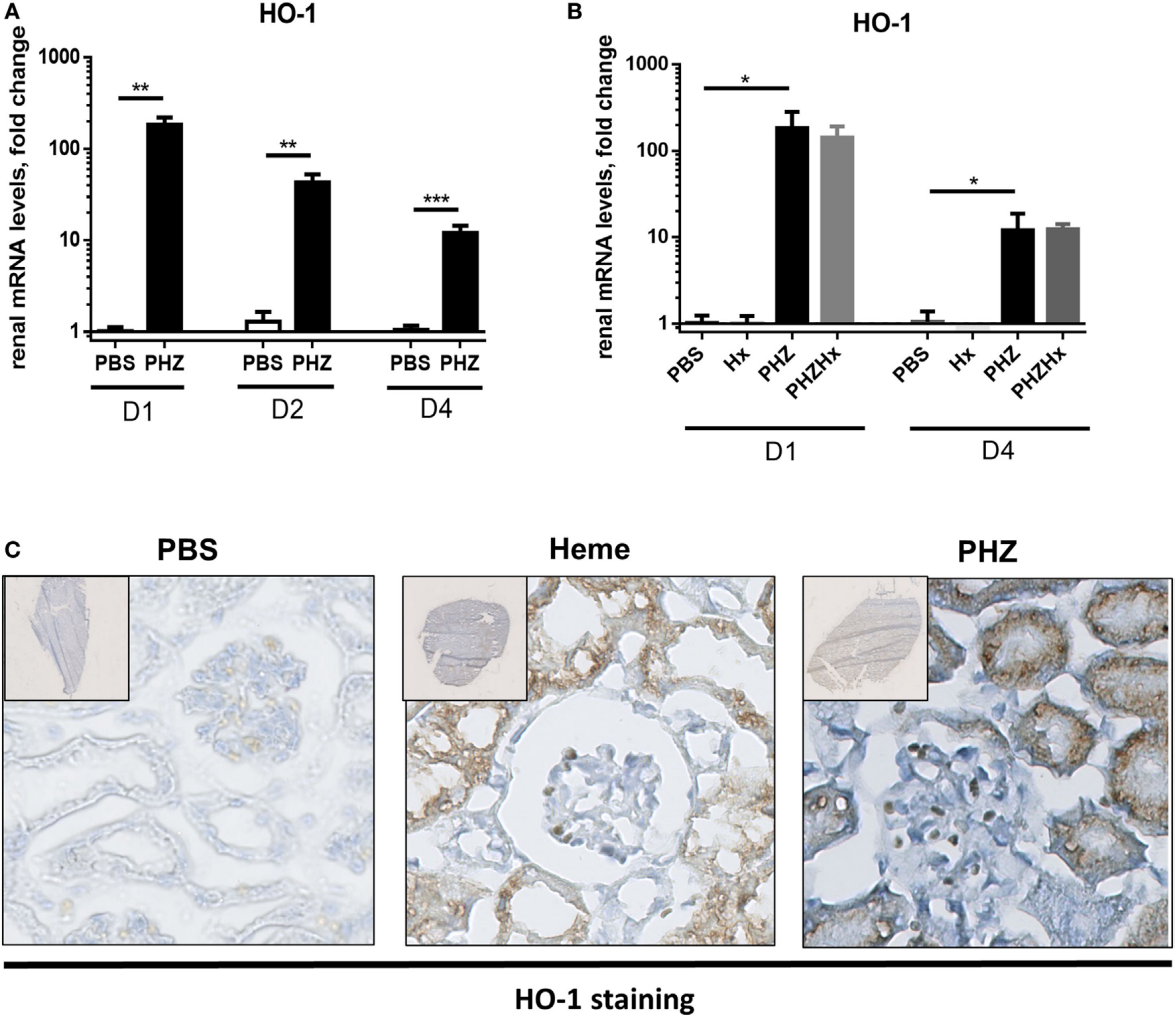
Heme oxygenase 1 (HO-1) expression induced in tubular kidneys in response to hemolysis. **(A)** Kinetics of mRNA levels in renal tissue of HO-1 at days 1, 2, and 4. ** *p* < 0.005, *** *p* < 0.001, and Mann–Whitney test. **(B)** HO-1 expression at days 1 and 4 was not modulated by hemopexin (Hx); * *p* < 0.05, Kruskal–Wallis with Dunn’s test for multiple pairwise comparisons. **(C)** Immunohistochemistry analysis of frozen kidney sections of mice (×20), injected with phenylhydrazine (PHZ), heme, or with the vehicle only at day 2. The staining for HO-1 appears in brown and nuclei in blue. Representative images from one out of three or five mice per group (staining performed in three independent experiments).

Strong upregulation of the cytoprotective enzyme HO-1 till day 4 at both gene (Figures 5A,B) and protein levels (Figure 5C) was detected in kidneys of PHZ-injected mice. Preinjection of Hx did not modify expression of HO-1 in PHZ-treated mice (Figure 5B). Treatment with heme also resulted in upregulation of this enzyme (Figure 5C). HO-1 staining was localized in the tubules. Immunohistochemistry revealed no HO-1 expression in PBS-treated mice (Figure 5C).

### Markers of Tubular Injury Were Found in the Urine of PHZ-Injected Mice

**Figure 6:**
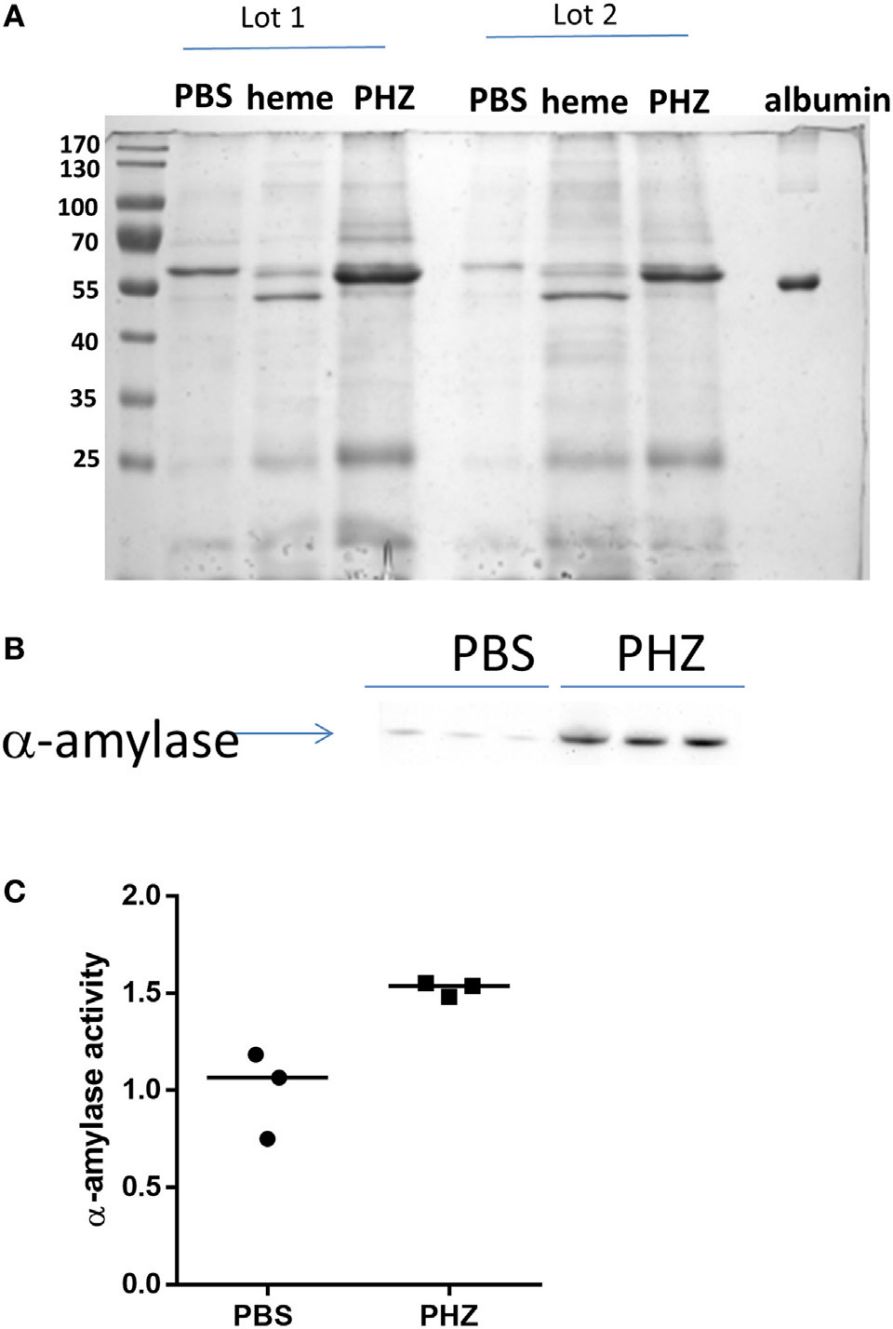
Phenylhydrazine (PHZ) triggered pancreatic α-amylase activity. **(A)** Proteinuria analysis by Coomassie blue staining of mice treated with PHZ, heme, or vehicle only. **(B)** Western blot analysis of α-amylase excreted in urines of mice treated with PHZ, heme, or vehicle only. **(C)** Comparison of α-amylase activity in urines from mice treated with PHZ or vehicle only. According to the manufacturer, urines were put in the presence of the substrate ethylidene-pNP-G7, which is cleaved in the presence of α-amylase, causing the release of a chromophore that can then be measured at OD = 405 nm.

To find out if the tubular necrosis and the oxidative and inflammatory stress in the kidney result in alteration of proteinuria, the profile of the urinary protein content was examined by electrophoresis (Figure 6A). When the 24-h urine samples were analyzed at equal volume, without normalization to creatinine, it revealed increased total protein content in the PHZ-injected mice (in agreement with the measurement of total protein). Nevertheless, when the urine was diluted to normalize to creatinine, the protein content was similar to the PBS-treated mice (not shown). Alteration of the renal endothelium could affect the glomerular microvasculature and the filtration function of the kidney. A distinction between glomerular and tubular proteinuria could be made in experimental models based on the profile of urine electrophoresis, appearance of low-molecular weight proteins (<60 kDa) suggesting tubular damage, while high-molecular weight proteins (>60 kDa) indicating glomerular one. No apparent major imbalance of the higher and lower molecular weight proteins was revealed by the electrophoresis, when normalization to creatinine was performed, but a tendency to higher level of low-molecular weight proteins was noted (not shown).

Nevertheless, when samples were deposited at equal volume (for qualitative analyses) several proteins that were not present in the PBS controls were specifically identified in the PHZ and heme samples. To identify these proteins and find out whether they reflect alteration of the tubular or glomerular function, a mass spectrometry analyses were performed by in-gel trypsin digestion of the proteins. Urine of PHZ-treated animals was positive for meprin-α, cytoskeletal keratins, α-1-antitrypsine and α-1-microglobulin (protein AMBP), while heme-treated mice urine was positive for meprin-α only (Table 1).

**Table 1:** Proteins, identified by mass spectrometry in each of the bands, excised from the gel of urinary electrophoresis.

MW kDa	Heme	PHZ	PBS	BSA
Band 200	Meprin A subunit alpha P28825	Meprin A subunit alpha P28825	Uromodulin Q91 × 17	
Band 75		Serum albumin P07724 Uromodelin Q91 × 17		
Band 70	Uromodulin Q91 × 17	Serotransferrin Q0921I1 Serum albumin P07724	Serotransferrin Q0921I1	
Band 65		Serotransferrin Q0921I1 Serum albumin P07724		
Band 60	Serum albumin P07724	Serum albumin P07724	Serum albumin P07724	Serum albumin P02769
Band 50–55	Pancreatic alpha-amylase P00688	Serum albumin P07724 Alpha-amylase 1 P00687		
Band 40	Pancreatic alpha-amylase P00688	Serum albumin P07724		
Band 38		Keratin, type-II cytoskeletal 79 Q8VED5 Alpha-1-antitrypsine 1-1 P07758 Alpha-1-antitrypsine 1-2 P22599 Alpha-1-antitrypsine 1-4 Q00897		
Band 35		Keratin, type-I cytoskeletal 42 Q6IFX2 Keratin, type-I cytoskeletal 14 Q61781		
Band 25	Kallikrein P15947	Kallikrein P15947 Protein AMBP Q07456	Kallikrein P15947 Serum albumin P07724	

*The numbers represent the UniProtKB ID of the protein*.

*PHZ, phenylhydrazine; BSA, bovine serum albumin; MW, molecular weight*.

### Hemolysis and Heme Injection-Induced Signs of Acute Pancreatitis

The gels revealed also a presence of a thick ~55-kD band, characteristic for the heme and to less extend to PHZ-injected mice, which was absent in the PBS controls (Figure 6A). Mass spectrometry analyses revealed that the protein is pancreatic α-amylase (Table 1). In-debt peptides analyses were performed to distinguish between salivary amylase and pancreatic amylase, confirming the presence of the pancreatic isoform of the enzyme (data not shown). This result was confirmed by western blot in the PHZ-injected group (Figure 6B).

Presence of pancreatic α-amylase in the urine could suggest an acute pancreatitis, caused by heme toxicity and hemolysis. To test this hypothesis, measurement of plasmatic pancreatic α-amylase was performed and these were found indeed increased in PHZ-treated animals as compared with controls (Figure 6C).

## Discussion

Here, we provide a detailed description of the renal phenotype of a mouse model of intravascular hemolysis, triggered by injection of PHZ. Hemolysis induced rapidly renal inflammation and mild tubular injury, which were largely heme-independent. In addition, we found signs of multiorgan injury, as revealed by major alterations of multiple metabolic parameters in mice, including acute pancreatitis in response to heme and PHZ.

Diverse experimental models of intravascular hemolysis have been described, but the most common one is drug-induced hemolysis with PHZ. Using this model, the protective role of hemoglobin- and heme-scavenging proteins, such as haptoglobin and Hx, as well as heme-degrading enzyme HO-1 function have been demonstrated (16, 18, 27, 28).

A main concern in this model is the specificity of the used drug. Indeed, PHZ is classically applied to trigger experimental erythrocytes lysis, but its effects on other cell types are poorly documented. Our results indicate that even high doses of PHZ do not cause endothelial cell activation and the observed expression of endothelial activation markers is, therefore, related to the hemolysis-derived products, rather than to direct effects of PHZ itself. *In vitro* high doses of PHZ (corresponding to the half of the final concentration injected *in vivo*) were cytotoxic to HUVEC, when exposed for 24 h in culture medium, containing only 20% fetal calf serum. *In vivo*, though, Caspase-3 activation in the kidneys was not detected and no vascular or glomerular damage was observed in PHZ-treated animals. The first hepatic passage and detoxification of the drug due to the i.p. injection and the high-protein content in the blood prevented direct toxicity to endothelium. Since erythrocytes are particularly susceptible to even low doses of PHZ, we could conclude that the major effects observed in the current model, at least in the kidney, are due to the hemolysis-derived products and less likely to direct effects of PHZ.

Kidneys are the primary route for hemoglobin clearance after saturation of the natural scavenging systems, and they are therefore highly susceptible to organ dysfunction during hemolysis. Renal lesions are described as major complications of hemolysis (29), but not all patients develop overt renal manifestations. Our histological work in the PHZ-injected mice revealed minor but significant tubular dilatation, suggestive of tubular necrosis. We observed an upregulation of sensitive markers for tubular injury, such as NGAL and Kim-1 (30), in agreement with other models of hemolysis, including old blood transfusion in mice (16) and guinea pigs (25). Persistent Kim-1 expression at day 4, when most other markers have returned to normal, indicated that the reparative process was rapid, yet incomplete at this time point. Multiple markers pointed at tubular injury. Increased expression of the proliferation marker Ki-67 is an indirect marker of tubular repair after injury. Gel electrophoresis of urinary proteins revealed also presence of (proximal) tubular damage protein markers, such as meprin-α (31) α-1-antitrypsine, renal keratins (32), and α-1-microglobulin (33), indicating tubular alterations. Interestingly, α-1-microglobulin is a heme-binding protein (34), which contributes to the clearance of heme and free radicals, released from Hb in extravascular fluids (35). Meprin-α is involved in the activation and degradation of inflammatory cytokines such as IL-1 and IL-6, respectively (36, 37). All these parameters demonstrate that tubular injury is one of the major lesions of hemolysis in the kidney in this model. This corroborates previous studies showing that injecting PHZ to mice (0.2-mg/g body weight) results in renal tissue damage 48 h later, revealed by reduced ^3^H-inulin clearance (38). Our results are also coherent with previously published hemolytic models of stored erythrocyte transfusion in mice (16) and guinea pigs (16, 25), which reported distorted renal tubule function as a primary cause for renal impairment during intravascular hemolysis (25).

Renal inflammation has rarely been studied in detail in hemolytic models. In PHZ-injected mice, we detected IL-6 as a marker of tissue inflammation, as previously reported in other hemolysis models (16). IL-6 is produced by activated macrophages as well as kidney-resident cells including podocytes, endothelial, mesangial, and tubular epithelial cells. IL-6 induces vasoconstriction and endothelial dysfunction, increases ROS production and tubular atrophy, and could thus contribute to renal injury during intravascular hemolysis. Heme and hemolysis (induced by PHZ at 0.1 mg/g, followed by a second injection 16 h later at 0.05 mg/g) triggered the processing and secretion of IL-1β by macrophages *via* NLRP3- and inflammasome-mediated signals (39). Nevertheless, in the kidneys of PHZ-treated mice, there was no evidence for immune cells infiltration by histology and immunofluorescence, explaining thus the lack of upregulation of IL-1β and TNF-α.

Taken together, our results indicate that kidneys respond to hemolysis through increased endothelial activation, since we found pro-inflammatory changes in renal endothelial cells, with upregulated expression of P-selectin, E-selectin, and ICAM-1. These adhesion molecules are thought to contribute to renal inflammation (38, 40–42) and vascular injury (25, 43). Inflammation resolved rapidly and these markers returned to baseline between days 2 and 4 after PHZ administration.

Detailed examination of metabolic parameters and renal function in PHZ-treated mice revealed extensive anemia, but only minor alterations of uremia, and no difference in parameters related to glomerular filtration at 48 h. We detected increased proteinuria and severe, transient oliguria (at 24 h). These may be explained by reduced water uptake. We also detected reduced food uptake, hence reduced excrements, with subsequently reduced body weight. Altogether, obtained results indicate that murine kidneys are relatively resistant to hemolysis-induced acute kidney injury. Local inflammation and tubular and vascular stress appeared on the first day after hemolysis but resolved rapidly. Therefore, extensive analyses of metabolic parameters must be performed in murine models of intravascular hemolysis, in order to determine to what extend renal injury is related to the alteration of kidney function, or merely to the altered overall health status of the anemic animals.

The poor overall health status of the PHZ- and heme-injected animals suggests that other organs may be more affected than kidneys. Indeed, we detected alteration of the mRNA level of these markers also in the liver and spleen, as expected from alternative hemolysis models (16). Moreover, analyses of urinary protein profiles revealed a surprising band of about 55 kDa in PHZ- and heme-injected mice, which was not present in PBS-injected controls. Mass spectrometry indicated that this band corresponds to pancreatic α-amylase, as confirmed by western blotting. The presence of pancreatic α-amylase in urine is not thought to be related to renal injury, but to acute pancreatitis (44). Acute pancreatitis in our PHZ- and heme-injected mice was confirmed by increases in plasmatic amylase. Early oxidative protein modifications and free radicals, as found in intravascular hemolysis in humans, contribute to functional impairment of the pancreas (45). Acute pancreatitis is a known complication of hemolytic diseases, such as SCD and autoimmune atypical hemolytic uremic syndrome (46, 47), and it was reported in a rat model of intravascular hemolysis involving a PHZ analog (48–50). In rats, pancreatic inflammation was made evident by increased TNF-α, IFN-γ, IL-18, ICAM-1, and MCP-1 levels subsequent to hemolysis (50). Aggravated pancreatic injury and inflammation were detected when the main heme-degrading enzyme, HO-1, was inhibited. Detailed analyses, beyond the scope of this study, will reveal which other organs may be affected the most by intravascular hemolysis.

We sought to determine the contribution of hemolysis-derived products other than heme, to the adverse effects of intravascular hemolysis. Heme is a well-known TLR-4 ligand (20, 51) that can activate cells (8, 20, 39, 43, 52, 53). Therefore, it was tempting to speculate that the renal injury in PHZ-treated mice resulted from massive heme release. Contrary to this speculation, we found that injection of heme alone resulted only in a slight increase in tubular injury markers, such as NGAL mRNA level and meprin-α in urine.

One may argue that the dose of heme injected was insufficient to trigger more significant kidney injury, or that part of it did not reach the circulation after intraperitoneal injection. Indeed, crystalline heme is poorly soluble in aqueous solutions and prone to aggregation upon injection *in vivo* (9). We could not measure free heme in plasma after heme administration, as it is technically very difficult to distinguish hemoglobin-bound, from protein-bound and protein-free heme. Indeed, the exact concentrations of free heme in hemolytic diseases remain debated. Heme tends to bind to a large number of circulating proteins (7) or remains associated with erythrocyte membrane microvesicles (52, 54). We tested the injection of purified Hx as a tool to investigate the contribution of free heme to renal inflammation. If heme drove stress gene overexpression, its scavenging by Hx would lead to decreased expression. The gene expression pattern we observed in PHZ-induced mice was not modified by Hx addition, despite the fact that Hx did reach the circulation (data not shown). These results are in line with the crucial role of hemoglobin for the renal injury (16). Indeed, in another model of hemolysis, haptoglobin but not Hx decreased NGAL, Kim-1, and IL-6 genes expression, as well as Ki-67 staining in kidneys (16). These results suggested that heme alone does not trigger major change in renal phenotype, but induces only mild and transient changes in metabolic parameters. One potential explanation could be the residual Hx in plasma and the rapid upregulation of HO-1. HO-1 is the principal physiological mechanism of heme degradation, and a defense mechanism against oxidative stress (35). Heme and PHZ treatment induced a potent upregulation of HO-1 in mouse kidney tubules, in agreement with previous observations (19, 55, 56). Therefore, the induction of HO-1 and its cytoprotective function may explain the rapid resolution of renal inflammation and the absence of severe acute kidney injury in our PHZ-injected mice (57–59). Moreover, despite its side effects, heme arginate was approved as a drug for treatment of acute porphyria (60).

In multiple studies, heme injection was used for tissue precon-ditioning, as a mean to enhance HO-1 activity in order to protect organs from subsequent challenges. This was studied in animal models (61–63) and used in human clinical trials for renal transplantation (64). On the other hand, heme promotes vaso-occlusions and contributes to the severity of crises in experimental models of SCD (20, 21, 65, 66). Hence, the potentially opposed effects of heme must be taken into account when choosing an experimental model in order to study intravascular hemolysis.

## Conclusion

Our results, added to the literature, converge to suggest that the injection of heme remains a method of choice for targeted, mechanistic studies, or as a tool to provoke vaso-occlusions in murine models of SCDs. However, PHZ-induced intravascular hemolysis bears several advantages over heme injection, as it emulates the pathological process of erythrocyte destruction, combined with hemoglobin and heme release. Also, it gives reproducible results and is easy to handle. PHZ administration, studied herein, may thus be preferred to study erythrocyte degradation in physiological processes and hemolytic diseases, to establish the benefits of HO-1-mediated therapy, or to investigate the effects of hemoglobin and heme scavengers.

## Ethics Statement

All experiments were conducted in accordance with the recommendations for the care and use of laboratory animals and with the approval APAFIS#3764-201601121739330v3 of the French Ministry of Agriculture. C57Bl/6 mice were from Charles River Laboratories (L’Arbresle, France).

## Author Contributions

Study design: LTR, NM, JDD. Perform research: NM, AG, MLF, SC, MD, TRR, RN, MR, SK, NB, TG, VL, FG, RD. Discussed the data: LTR, JDD, OBB, RD, NM, AG, SC, MLF, PH, MR, SM, VL, FG, OM, MF, MD, TRR. LR, NM and JDD wrote the manuscript. All authors approved the submission.

## Conflict of Interest Statement

NB, TG, and SM are employees of CSL Behring. LR receives research funding from CSL Behring. The remaining authors declare no conflict of interest.
